# Supplementary material for: Use of Next-Generation Sequencing to Support the Diagnosis of Familial Interstitial Pneumonia
Source: Genes (Basel). 2023 Jan 27;14(2):326. doi: 10.3390/genes14020326 (PMC9957248; doi:10.3390/genes14020326)
Supplement: Supplementary file 1 [file genes-14-00326-s001.zip › genes-2107115-SI.pdf]

Supplementary material

Table S1. Gene panel analysed by NGS in each patient.

Patient	Gene panel	Genetic variants detected
#1	ABCA3, DKC1, MUC5B, NKX2-1, RTEL1, SFTPA1, SFTPA2, SFTPB, SFTPC, SFTPD, TERC, TERT and TINF2	SFTPA2 c.135C>T
#2	ABCA3, CSF2RA, CSF2RB, DMBT1, MUC5B, SFTPA2, SFTPB, SFTPC, SFTPD, TERC and TERT	TERT c.1072C>T
#3	ABCA3, DKC1, DMBT1, MUC5B, NKX2-1, RTEL1, SFTPA1, SFTPA2, SFTPB, SFTPC, SFTPD, TERC, TERT and TINF2	DMBT1 c.3052T>A
#4	ABCA3, CSF2RA, CSF2RB, DMBT1, MUC5B, SFTPA2, SFTPB, SFTPC, SFTPD, TERC and TERT	ABCA3 c.694C>T
#5	ABCA3, DKC1, MUC5B, NKX2-1, RTEL1, SFTPA1, SFTPA2, SFTPB, SFTPC, SFTPD, TERC, TERT and TINF2	RTEL1 c.3775_3776del
#6	ABCA3, CSF2RA, CSF2RB, DKC1, MUC5B, NKX2-1, RTEL1, SFTPA1, SFTPA2, SFTPB, SFTPC, SFTPD, TERC, TERT and TINF2	MUC5B c.9563C>T
#7	ABCA3, CSF2RA, CSF2RB, DKC1, MUC5B, NKX2-1, PARN, RTEL1, SFTPA1, SFTPA2, SFTPB, SFTPC, SFTPD, TERC, TERT and TINF2	TINF2 c.1285C>A
#8	ABCA3, DKC1, MUC5B, NKX2-1, RTEL1, SFTPA1, SFTPA2, SFTPB, SFTPC, SFTPD, TERC, TERT and TINF2	RTEL1 c.3775_3776del
#9	ABCA3, CSF2RA, CSF2RB, DKC1, MUC5B, NKX2-1, PARN, RTEL1, SFTPA1, SFTPA2, SFTPB, SFTPC, SFTPD, TERC, TERT and TINF2	TERT c.2701C>T ABCA3 c.2026G>A c.1417G>A
#10	ABCA3, CSF2RA, CSF2RB, DKC1, MUC5B, NKX2-1, PARN, RTEL1, SFTPA1, SFTPA2, SFTPB, SFTPC, SFTPD, TERC, TERT and TINF2	MUC5B c.1855C>T
#11	ABCA3, CSF2RA, CSF2RB, DKC1, MUC5B, NKX2-1, PARN, RTEL1, SFTPA1, SFTPA2, SFTPB, SFTPC, SFTPD, TERC, TERT and TINF2	PARN c.1500T>G
#12	ABCA3, CSF2RA, CSF2RB, DKC1, MUC5B, NKX2-1, PARN, RTEL1, SFTPA1, SFTPA2, SFTPB, SFTPC, SFTPD, TERC, TERT and TINF2	PARN c.24dup
#13	ABCA3, DKC1, MUC5B, NKX2-1, RTEL1, SFTPA1, SFTPA2, SFTPB, SFTPC, SFTPD, TERC, TERT and TINF2	RTEL1 c.2672C>T
#14	ABCA3, CSF2RA, CSF2RB, DMBT1, MUC5B, SFTPA2, SFTPB, SFTPC, SFTPD, TERC and TERT	Negative
#15	ABCA3, DKC1, MUC5B, NKX2-1, RTEL1, SFTPA1, SFTPA2, SFTPB, SFTPC, SFTPD, TERC, TERT and TINF2	Negative
#16	ABCA3, DKC1, MUC5B, NKX2-1, RTEL1, SFTPA1, SFTPA2, SFTPB, SFTPC, SFTPD, TERC, TERT and TINF2	Negative
#17	ABCA3, CSF2RA, CSF2RB, DKC1, MUC5B, NKX2-1, PARN, RTEL1, SFTPA1, SFTPA2, SFTPB, SFTPC, SFTPD, TERT and TINF2	Negative
#18	ABCA3, CSF2RA, CSF2RB, DKC1, MUC5B, NKX2-1, RTEL1, SFTPA1, SFTPA2, SFTPB, SFTPC, SFTPD, TERC, TERT and TINF2	Negative

#19	ABCA3, CSF2RA, CSF2RB, DMBT1, MUC5B, SFTPA1, SFTPA2, SFTPB, SFTPC, SFTPD, TERC and TERT	Negative
#20	ABCA3, CSF2RA, CSF2RB, DKC1, MUC5B, NKX2-1, RTEL1, SFTPA1, SFTPA2, SFTPB, SFTPC, SFTPD, TERC, TERT and TINF2	Negative
